# Social effects on age-related and sex-specific immune cell profiles in a wild mammal

**DOI:** 10.1098/rsbl.2020.0234

**Published:** 2020-07-15

**Authors:** Sil H. J. van Lieshout, Elisa P. Badás, Michael W. T. Mason, Chris Newman, Christina D. Buesching, David W. Macdonald, Hannah L. Dugdale

**Affiliations:** 1 School of Biology, Faculty of Biological Sciences, University of Leeds, Leeds LS2 9JT, UK; 2 Groningen Institute for Evolutionary Life Sciences, University of Groningen, 9747 AG Groningen, The Netherlands; 3 Wildlife Conservation Research Unit, Department of Zoology, University of Oxford, The Recanati-Kaplan Centre, Abingdon, Oxfordshire OX13 5QL, UK

**Keywords:** European badger, social effects, innate immunity, adaptive immunity, neutrophil, lymphocyte

## Abstract

Evidence for age-related changes in innate and adaptive immune responses is increasing in wild populations. Such changes have been linked to fitness, and knowledge of the factors driving immune response variation is important for understanding the evolution of immunity. Age-related changes in immune profiles may be owing to factors such as immune system development, sex-specific behaviour and responses to environmental conditions. Social environments may also contribute to variation in immunological responses, for example, through transmission of pathogens and stress arising from resource and mate competition. Yet, the impact of the social environment on age-related changes in immune cell profiles is currently understudied in the wild. Here, we tested the relationship between leukocyte cell composition (proportion of neutrophils and lymphocytes [innate and adaptive immunity, respectively] that were lymphocytes) and age, sex and group size in a wild population of European badgers (*Meles meles*). We found that the proportion of lymphocytes in early life was greater in males in smaller groups compared to larger groups, but with a faster age-related decline in smaller groups. By contrast, the proportion of lymphocytes in females was not significantly related to age or group size. Our results provide evidence of sex-specific age-related changes in immune cell profiles in a wild mammal, which are influenced by the social environment.

## 1. Introduction

The immune system involves multiple mechanisms that protect the host against pathogens [1]. The functioning of the immune system is related to sex [2,3], changes throughout life [4–9] and has been linked to mortality in the wild [9]. Investigation of how such factors drive variation in immune responses is important for understanding the evolution of immunity.

The immune system principally comprises two components: innate and adaptive immunity [1]. The innate immune response is the first defence against pathogens, involving phagocytic cells (e.g. neutrophils, macrophages and dendritic cells) that detect antigens and produce cytokines, which trigger other parts of the immune system [10–14]. The activation of adaptive immunity includes the cell-mediated immune response, with the stimulation of T lymphocytes and humoral immunity, and activated B lymphocytes that differentiate to produce immunoglobulins against specific antigens [13,15]. The relative components of innate and adaptive immunity are therefore often reflected in the neutrophil–lymphocyte ratio, respectively [16–19].

The adaptive immune system generally undergoes an age-related decline in performance, i.e. immunosenescence, and evidence for this process has been emerging in wild populations [4–9]. By contrast, the innate immune response is usually maintained, or even enhanced with age [4–9]. This enhanced innate immune response can be a consequence of overstimulation of the immune system, owing to a reduced T cell repertoire and bias towards CD8+ effector memory cells, leading to chronic inflammation and accelerated immunosenescence, as seen in humans [20,21].

The innate and adaptive immune responses, mediated by genes and hormones, are sex-specific [2,3]. For example, in the human innate immune response, males typically have higher neutrophil and macrophage phagocytic activity than females [22,23], whereas in the adaptive immune response, females typically have stronger antibody responses, higher basal immunoglobulin levels and more B cells than males [22,24]. Such sex differences in immune responses may become exacerbated with age [3,25]. For example, male Soay sheep (*Ovis aries*) exhibit steeper sex-specific changes in leukocyte cell composition with age [26]. However, such changes may be species-specific since no sex differences in leukocyte cell composition with age were detected in roe deer (*Capreolus capreolus*; [5]).

Social stress is also emerging as a potential driver of variation in immune responses in the wild [27–29], with stress being reflected in the neutrophil–lymphocyte ratio [30]. Gregarious individuals often experience greater stress owing to more social interactions or increased mate competition [28,31,32]. Testosterone can have a suppressive effect on the immune system ([33,34], but see [35]), and polygynous males have more circulating testosterone than conspecific females or monogamous males. Thus, the social system and the environment can have a sex-specific effect on immune cell profiles. Social individuals may also experience greater costs of pathogen exposure owing to group-living, compared with solitary individuals [29]. For example, greater early-life exposure to pathogen variety and intensity within social groups could prime the immune system and result in enhanced later-life immunity but with the risk of later-life auto-immunity [36,37]. However, to date, there has been no clear evidence for the effects of the social environment on sex-specific immune cell profiles and their age-related changes in the wild.

Here, we use blood samples collected from a wild population of European badgers (*Meles meles*; hereafter ‘badger') to explore longitudinal changes in sex-specific immune cell profiles in relation to social conditions. We quantify the relative components in the immune system through the proportion of neutrophils and lymphocytes that are lymphocytes (henceforth ‘proportion of lymphocytes'), which reflects the relative balance between innate and adaptive immunity [16–19]. Specifically, we test whether the proportion of lymphocytes: (i) changes with age, (ii) exhibits sex differences and (iii) is linked to group size.

## 2. Methods

### (a) Study species and data collection

We conducted this study in Wytham Woods, Oxfordshire, UK (51°46′24″ N, 1°20′04″ W), a 424 ha semi-natural woodland surrounded by mixed arable pasture [38]. The resident high-density badger population (mean ± s.e. = 36 ± 3 badgers/km^2^; [39]) consists of large mixed-sex social groups (mean group size = 11, range = 2–29; [40]). Badgers have a polygynandrous mating system with high rates of extra-group paternity [41,42], where males exhibit seasonal peaks in testosterone levels [43,44]. Badgers are exposed to pathogens, such as coccidia, that negatively impact development and cause juvenile mortality [45–47].

Trapping was undertaken three times per year, for three consecutive days per social group in 2017 and 2018. Trapped badgers were anaesthetized using an intra-muscular injection of 0.2 ml ketamine hydrochloride per kg body weight [48]. Individuals were identified by a unique tattoo number on the left inguinal region, with capture date, social group affiliation and sex recorded. Age was determined as the difference between capture date and the 14th of February in the birth year, since implantation and parturition dates are highly synchronous in badgers [49–51]. Badgers first caught as adults were aged through tooth wear (scale 1–5), where a score of 2 typically indicates a 1-year-old adult [52]. Blood was collected through jugular venipuncture into vacutainers with EDTA anticoagulant. Badgers were released at their setts, after full recovery from anaesthesia. Additionally, bait-marking was conducted periodically to delimit social groups [53] and calculate group sizes using dispersal rules (see electronic supplementary material).

Immediately after blood collection, one drop of blood was smeared on a microscope slide. Slides were air-dried for 1 h then stained using Kwik-Diff (Thermo Scientific, Manchester, UK) according to the manufacturer's protocol. Leukocyte cell counts were conducted by the same observer (blind to group size and sex) by counting 100 cells per slide (4 repeats per slide, not consecutively to avoid bias; *n* *=* 82 slides, 23 individuals; 9 females, 14 males), at 40 x magnification using the battlement technique [54]. Cells were identified as neutrophils, eosinophils and basophils (i.e. granulocytes) or lymphocytes and monocytes (i.e. agranulocytes; [55]). Basophils (< 0.1%), eosinophils (1.4%) and monocytes (3.4%) were rarely observed, thus we only used neutrophils and lymphocytes to calculate the lymphocyte proportion from these data [56].

### (b) Statistical analyses

Statistical analyses were conducted in R. 3.3.1 [57], using parametric bootstrapping (*n* = 5000) as a robust method to determine significance of predictors and 95% confidence intervals in *lme4* 1.1–14 [58–60]. The mixed model had a binomial error distribution (link = logit), as recommended with a proportional response variable [61] (proportion of lymphocytes), with an offset to account for the number of cells counted per slide (*n* = 7 repeats, 5 slides, where a total of 100 neutrophils and lymphocytes were counted on a slide). Models were run separately for males and females to test for a sex-specific effect with both age [3,26] and group size [62]. To ensure that separating our models by sex did not alter out conclusions (e.g. owing to reduced statistical power), we also ran a model with both sexes included.

We first compared the fit of the relationship between age versus logarithmic age and the proportion of lymphocytes using AICc values; a negative logarithmic pattern was best supported in the full dataset (ΔAICc = −3.8) and males (ΔAICc = −2.3), but with little difference in females (ΔAICc = 0.2). Logarithmic age was therefore included in the mixed model analysing the full dataset and in the separate models for males and females, but the female models were also checked with linear age. We then used AICc to determine *a priori* whether interactions between age, group size and sex (full dataset) and between age and group size (sex-specific datasets) should be included (electronic supplementary material, table S1). When multiple models were plausible (ΔAICc < 7; [63]) and the interaction was non-significant, we re-ran the model without the interaction to also accurately test the first-order effects. We also included season, year and body condition index (log_10_weight/log_10_body length; [44,64]) as fixed effects since these affect immune cell concentrations [65–67]. Cohort, social group, slide nested within individual ID and observation (for each unique measure to account for overdispersion [68]) were included as random effects.

## 3. Results

**Figure 1.**
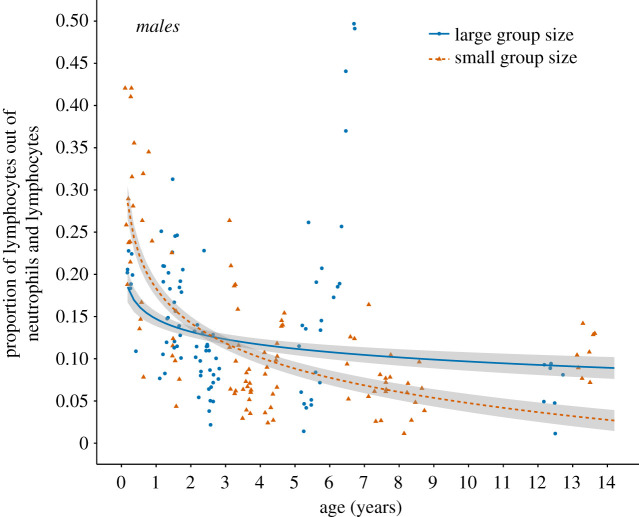
The interplay between age and group size on the proportion of neutrophils and lymphocytes that were lymphocytes for males. Raw data points are shown. Group size was modelled as a continuous variable in the mixed model, but for visualization is shown in small (range = 1–9; *n* *=* 99 repeats; 25 slides; 9 individuals; brown triangles and dashed line) and large (range = 10–16; *n* = 96 repeats; 24 slides; 8 individuals; blue circles and solid line) groups. Three males were part of a large group at one time point and a small group at another time point, hence total sample size differs from the methods section (§2). Fitted lines represent the model prediction for age interacting with group size, with associated 95% confidence intervals as shaded areas.

In males, we found an interaction between age and group size on the proportion of lymphocytes (table 1). Males living in smaller groups had a higher proportion of lymphocytes in early life, which declined more steeply with age than in males in larger groups, such that the proportion of lymphocytes decreased with age by 50% for males in larger groups compared to 80% for males in smaller groups (figure 1 and table 1). By contrast, for females, the proportion of lymphocytes did not differ significantly according to group size or age (table 2 and electronic supplementary material, table S2), or when using linear age (electronic supplementary material, tables S3 and S4). The full dataset showed an interaction between age, group size and sex on the proportion of lymphocytes (electronic supplementary material, table S5), indicating an interaction between age and group size that differs between males and females, thus providing similar results to the models analysing the sexes separately.

**Table 1.** Parameter estimates and 95% confidence intervals of fixed effects from a mixed model and subsequent parametric bootstrapping testing age and group size effects on the proportion of neutrophils and lymphocytes that were lymphocytes in male European badgers. *β* = direction and magnitude of effect, s.e. = standard error, 95% CI = 95% confidence intervals; reference terms in brackets = reference level for factors; × = interaction. Significant parameters (95% CI does not overlap zero) are in italics. Random effect estimates (variance): individual ID (<1.000 × 10^−12^), slide nested in individual ID (1.378 × 10^−1^), social group (1.979 × 10^−2^), cohort (<1.000 × 10^−12^), observation (1.080 × 10^−1^).

parameter (reference level)	*β*	s.e.	95% CI
*intercept*	−2.325	0.127	−2.570 to −2.073
*log age*	**−***0.211*	*0.095*	**−***0.403 to* **−***0.015*
*group size*	*0.220*	*0.087*	*0.050 to 0.388*
year (2017)			
* 2018*	*0.421*	*0.137*	*0.148 to 0.693*
season (spring)			
summer	−0.046	0.131	−0.310 to 0.215
* autumn*	*0.617*	*0.224*	*0.156 to 1.069*
*body condition index*	**−***0.255*	*0.097*	**−***0.446 to* **−***0.065*
*log age* × *group size*	*0.202*	*0.052*	*0.101 to 0.304*

**Table 2.** Parameter estimates and 95% confidence intervals of fixed effects from a mixed model and subsequent parametric bootstrapping testing age and group size effects on the proportion of neutrophils and lymphocytes that were lymphocytes in female European badgers. *β* = direction and magnitude of effect, s.e. = standard error, 95% CI = 95% confidence intervals; reference terms in brackets = reference level for factors; × = interaction. Significant parameters (95% CI does not overlap zero) are in italics. Random effect estimates (variance): individual ID (4.310 × 10^−2^), slide nested in individual ID (1.879 × 10^−1^), social group (<1.000 × 10^−12^), cohort (<1.000 × 10^−12^), observation (1.206 × 10^−1^).

parameter (reference level)	*β*	s.e.	95% CI
*intercept*	−2.284	0.188	−2.661 to −1.911
log age	−0.078	0.151	−0.386 to 0.214
group size	−0.107	0.115	−0.344 to 0.122
year (2017)			
2018	−0.017	0.211	−0.447 to 0.405
season (spring)			
summer	0.137	0.194	−0.228 to 0.527
autumn	0.558	0.317	−0.038 to 1.203
body condition index	−0.262	0.143	−0.551 to 0.015
log age × group size	−0.015	0.117	−0.245 to 0.210

## 4. Discussion

We found that social conditions (i.e. group size) have sex-specific effects on individual immune cell profiles with age. In male badgers in larger groups, early life exposure to a greater diversity, or higher intensity, of pathogens or greater stress associated with resource or mate competition could have possibly led to a stronger bias toward innate over adaptive immune cell profiles with age. Male badgers grow to maturity faster than females, resulting in a slight sexual dimorphism, and male growth is predominantly affected by social factors, whereas weather conditions predominantly affect female development [69]. According to the ‘hygiene-hypothesis' [37,70], early life exposure to pathogens could alleviate the detrimental consequences of increased pathogen pressure in later life and thus slow age-related changes in immune cell profiles. In smaller groups, lower exposure to pathogens in early life could have the opposite effect [71,72], accelerating changes in immune cell profiles with age. Moreover, if fewer conspecifics share the pathogen burden, this could lead to a stronger pressure on the immune response and rapid changes in the proportion of lymphocytes. Indeed, we found that the proportion of lymphocytes in early life was greater in male badgers living in smaller social groups, but with a steeper age-related decline. There was no significant effect in females. This is supported by a previous study in this same population showing that coccidiosis caused by *Eimera melis* has a more severe effect on male badger cub development [45]. Thus, the greater proportion of neutrophils to lymphocytes that we observed in males in early life, compared to females, could reflect their greater immune response to juvenile coccidiosis.

We also found a relative decrease in the proportion of lymphocytes with age in males but not females. Possibly, female badgers develop a stronger immune response against pathogens in early life, as observed in Soay sheep, where males had a steeper decline in lymphocyte proportion with age than did females [26]. Male badgers, given the polygynandrous mating system, have high testosterone levels [43], particularly compared to monogamous species [44], which may lead to immunosuppression and stronger decreases in adaptive immunity (i.e. lymphocytes) with age ([33,34], but see [35]). The potentially immuno-suppressive effect of testosterone in male badgers accords with sex-specific responses to environmental conditions and associated sex differences in immune responses seen in other species [2,3].

The greatest changes in immune cell profiles in males occurred in early life, when the immune response is developing. Early-life changes may have arisen owing to there being quantitatively fewer acquired immunity cells, or more innate cells being produced. The later-life decrease in the proportion of lymphocytes with age seen in this study has been associated with age-related reduction in thymus size in humans [73,74], accompanied by lower numbers of naïve T cells [75] and CD4^+^ T and CD8^+^ subpopulations with age, which has detrimental implications for effective immune responses to new antigens [10,76–80]. Alternatively, innate immune mechanisms may become more active with age through increased production of pro-inflammatory cytokines [81]. Such low-grade chronic inflammation in older individuals has detrimental effects on health and contributes to senescence and the development of age-related pathologies [21].

While we cannot provide direct evidence of immunosenescence, as we measured the proportion of lymphocytes rather than the absolute number of leukocytes per unit volume of blood, the relative decrease in adaptive immune cells and increase in innate immune cells that we detected with age accords with previous studies in the wild [4–6]. Furthermore, understanding the changes in immune cell profiles with age in mammals is important for the interpretation of leukocyte telomere dynamics [52]. Since granulocytes (e.g. neutrophils) have longer telomeres than agranulocytes (e.g. lymphocytes) in humans and baboons [82,83], apparent changes in telomere length with age could be owing to a changing leukocyte cell composition, or selective loss of leukocytes, with age, leading to spurious inferences on telomere shortening.

We were unable to sample individuals until at least three months of age owing to welfare legislation (Protection of Badgers Act, 1992), and thus we cannot rule out the possibility of selective disappearance of individuals with poor innate immune responses, potentially linked to coccidiosis mortality [45]. Additionally, while we provide evidence of age-related changes in one immune parameter (i.e. leukocyte cell composition), immunity is complex and future studies should analyse multiple immune markers (e.g. specific antibodies, inflammatory parameters) together to understand trade-offs and drivers of variation in immune responses. Nonetheless, our results indicate that age-related changes in immune profiles are associated with the social environment and these effects differ between the sexes.

## Supplementary Material

Group_size_estimation_and_supp_tables

## Data accessibility

Data are available on Dryad (https://dx.doi.org/10.5061/dryad.2547d7wmx) [56] and the R code is archived at GitHub (https://github.com/DugdaleResearchGroup/Leukocyte).
