# Supplementary material for: Social effects on age-related and sex-specific immune cell profiles in a wild mammal
Source: Biol Lett. 2020 Jul 15;16(7):20200234. doi: 10.1098/rsbl.2020.0234 (PMC7423055; doi:10.1098/rsbl.2020.0234)
Supplement: Group_size_estimation_and_supp_tables [file rsbl20200234supp1.pdf]

Social effects on age-related and sex-specific immune cell profiles in a wild mammal

Sil H.J. van Lieshout, Elisa P. Badás, Michael W.T. Mason, Chris Newman, Christina D. Buesching,

David W. Macdonald & Hannah L. Dugdale

Group size estimation:

Group sizes were determined by the number of individuals (cubs and adults) that were present in a social group in a given year. Given high natal philopatry (75.8%), low permanent dispersal rates (19.1%), and high levels of inter-group movements leading to extra-group paternity in badgers [1], individuals (*n* = 1726) were assigned as a resident of a social group each year, according to the following rules adapted from [1, 2]:

1. Badgers first caught as cubs (*n* = 1241) were considered resident in the social group they were first caught, until they subsequently satisfied dispersal rules or were considered dead.
2. Badgers first caught as adults (*n* = 490) were assigned to their lifetime modal social group, until dispersal rules applied. If an individual was captured equally between two groups (*n* = 29), they were assigned to the social group they were initially captured in until dispersal rules applied.
3. Dispersal rules were satisfied when the two most recent captures of an individual (>30 days apart), as well as 1 of 2 captures before, were made in a different social group than the current residential social group. Individuals were resident in the new social group until dispersal rules applied again.

The number of individuals per social group were then calculated as the sum of individuals present in the social group in a given year.

Table S1: Comparison of models, through AICc, with and without the interaction between age, sex and group size using the full dataset and the age and group size interaction using the sex-specific datasets.

	AICc	ΔAICc
**Model 1**		
With interaction between log age * sex * group size	125450.5	-2.8
No interaction between log age * sex * group size	125453.3	
**Model 2 (males)**		
With interaction between log age * group size	75987.4	-9.8
No interaction between log age * group size	75997.2	
**Model 3 (females)**		
With interaction between log age * group size	49477.8	2.5
No interaction between log age * group size	49475.3	
**Model 3 (females)**		
With interaction between linear age * group size	49477.6	2.1
No interaction between linear age * group size	49475.5	

Table S2: Parameter estimates and 95% confidence intervals of fixed effects from a mixed model and subsequent parametric bootstrapping testing age and group size effects (without their interaction) on the proportion of neutrophils and lymphocytes that are lymphocytes in female European badgers. β = direction and magnitude of effect, s.e. = standard error, 95% CI = 95% confidence intervals; reference terms in brackets = reference level for factors. Significant parameters (95% CI does not overlap zero) are in bold.

**Parameter (reference level)**	**β**	**s.e.**	**95% CI**
Intercept	-2.276	0.183	-2.628 to -1.928
Log age	-0.076	0.150	-0.381 to 0.225
Group size	-0.107	0.114	-0.350 to 0.115
Year (2017)			
2018	-0.025	0.201	-0.418 to 0.378
Season (Spring)			
Summer	0.138	0.193	-0.237 to 0.520
Autumn	0.547	0.313	-0.063 to 1.170
Body condition index	-0.259	0.144	-0.542 to 0.032

Random effect estimates (variance): Individual ID ($4.659*10^{-2}$), Slide nested in individual ID ($1.858*10^{-1}$), Social group ($<1.000*10^{-12}$), Cohort ($<1.000*10^{-12}$), Observation ($1.207*10^{-1}$)

Table S3: Parameter estimates and 95% confidence intervals of fixed effects from a mixed model and subsequent parametric bootstrapping testing age and group size effects on the proportion of neutrophils and lymphocytes that are lymphocytes in female European badgers. β = direction and magnitude of effect, s.e. = standard error, 95% CI = 95% confidence intervals; reference terms in brackets = reference level for factors; * = interaction. Significant parameters (95% CI does not overlap zero) are in bold.

**Parameter (reference level)**	**β**	**s.e.**	**95% CI**
Intercept	-2.265	0.201	-2.666 to -1.880
Linear age	0.028	0.144	-0.258 to 0.315
Group size	-0.093	0.117	-0.323 to 0.136
Year (2017)			
2018	-0.100	0.204	-0.505 to 0.315
Season (Spring)			
Summer	0.165	0.188	-0.212 to 0.542
Autumn	0.494	0.316	-0.127 to 1.140
Body condition index	-0.259	0.132	-0.524 to 0.001
Linear age * Group size	0.067	0.103	-0.138 to 0.265

Random effect estimates (variance): Individual ID ($1.211*10^{-2}$), Slide nested in individual ID ($1.743*10^{-1}$), Social group ($<1.000*10^{-12}$), Cohort ($6.032*10^{-2}$), Observation ($1.207*10^{-1}$)

Table S4: Parameter estimates and 95% confidence intervals of fixed effects from a mixed model and subsequent parametric bootstrapping testing age and group size effects (without their interaction) on the proportion of neutrophils and lymphocytes that are lymphocytes in female European badgers. β = direction and magnitude of effect, s.e. = standard error, 95% CI = 95% confidence intervals; reference terms in brackets = reference level for factors. Significant parameters (95% CI does not overlap zero) are in bold.

**Parameter (reference level)**	**β**	**S.E.**	**95% CI**
Intercept	-2.293	0.191	-2.670 to -1.927
Linear age	0.011	0.133	-0.252 to 0.273
Group size	-0.094	0.115	-0.321 to 0.138
Year (2017)			
2018	-0.061	0.199	-0.453 to 0.338
Season (Spring)			
Summer	0.150	0.190	-0.228 to 0.530
Autumn	0.543	0.317	-0.069 to 1.198
**Body condition index**	**-0.279**	**0.129**	**-0.538 to -0.034**

Random effect estimates (variance): Individual ID ($1.131*10^{-2}$), Slide nested in individual ID ($1.843*10^{-1}$), Social group ($<1.000*10^{-12}$), Cohort ($4.444*10^{-2}$), Observation ($1.207*10^{-1}$)

Table S5: Parameter estimates and 95% confidence intervals of fixed effects from a mixed model and subsequent parametric bootstrapping testing age, sex and group size effects on the proportion of neutrophils and lymphocytes that are lymphocytes in European badgers. β = direction and magnitude of effect, s.e. = standard error, 95% CI = 95% confidence intervals; reference terms in brackets = reference level for factors; * = interaction. Significant parameters (95% CI does not overlap zero) are in bold.

**Parameter (reference level)**	**β**	**s.e.**	**95% CI**
Intercept	-2.360	0.122	-2.593 to -2.125
Log age	-0.108	0.104	-0.313 to 0.098
Sex (female)	0.115	0.121	-0.124 to 0.349
Group size	-0.152	0.099	-0.344 to 0.042
**Year (2017)**			
**2018**	**0.258**	**0.119**	**0.025 to 0.496**
Season (Spring)			
Summer	0.045	0.116	-0.186 to 0.278
**Autumn**	**0.628**	**0.196**	**0.251 to 0.995**
**Body condition index**	**-0.275**	**0.082**	**-0.432 to -0.114**
Log age * Sex (female)	-0.060	0.118	-0.293 to 0.172
Log age * Group size	-0.096	0.106	-0.302 to 0.115
**Sex (female) * Group size**	**0.392**	**0.124**	**0.146 to 0.637**
**Log age * Sex (female) * Group size**	**0.295**	**0.118**	**0.060 to 0.533**

Random effect estimates (variance): Individual ID ($1.223*10^{-2}$), Slide nested in individual ID ($1.821*10^{-1}$), Social group ($<1.000*10^{-12}$), Cohort ($7.078*10^{-9}$), Observation ($1.131*10^{-1}$)

References

1. Macdonald DW, Newman C, Buesching CD, Johnson PJ. 2008 Male-biased movement in a high-density population of the Eurasian badger (*Meles Meles*). *J. Mammal.* **89**, 1077-1086. (doi:10.1644/07-Mamm-a-185.1)

2. Annavi G, Newman C, Dugdale HL, Buesching CD, Sin YW, Burke T, Macdonald DW. 2014 Neighbouring-group composition and within-group relatedness drive extra-group paternity rate in the European badger (*Meles meles*). *J. Evol. Biol.* **27**, 2191-2203. (doi:10.1111/jeb.12473)
